# Transcriptomic analysis reveals key transcription factors associated to drought tolerance in a wild papaya (*Carica papaya*) genotype

**DOI:** 10.1371/journal.pone.0245855

**Published:** 2021-01-29

**Authors:** Humberto Estrella-Maldonado, Amaranta Girón Ramírez, Gabriela Fuentes Ortiz, Santy Peraza-Echeverría, Octavio Martínez-de la Vega, Elsa Góngora-Castillo, Jorge M. Santamaría

**Affiliations:** 1 Centro de Investigación Científica de Yucatán A.C., Mérida, Yucatán, México; 2 Independent Researcher, Mérida, Yucatán, México; 3 Langebio, Irapuato, Guanajuato, México; ICAR-Indian Institute of Agricultural Biotechnology, INDIA

## Abstract

Most of the commercial papaya genotypes show susceptibility to water deficit stress and require high volumes of irrigation water to yield properly. To tackle this problem, we have collected wild native genotypes of *Carica papaya* that have proved to show better physiological performance under water deficit stress than the commercial cultivar grown in Mexico. In the present study, plants from a wild *Carica papaya* genotype and a commercial genotype were subjected to water deficit stress (WDS), and their response was characterized in physiological and molecular terms. The physiological parameters measured (water potential, photosynthesis, Fv/Fm and electrolyte leakage) confirmed that the papaya wild genotype showed better physiological responses than the commercial one when exposed to WDS. Subsequently, RNA-Seq was performed for 4 cDNA libraries in both genotypes (susceptible and tolerant) under well-watered conditions, and when they were subjected to WDS for 14 days. Consistently, differential expression analysis revealed that after 14 days of WDS, the wild tolerant genotype had a higher number of up-regulated genes, and a higher number of transcription factors (TF) that were differentially expressed in response to WDS, than the commercial genotype. Thus, six TF genes (CpHSF, CpMYB, CpNAC, CpNFY-A, CpERF and CpWRKY) were selected for further qRT-PCR analysis as they were highly expressed in response to WDS in the wild papaya genotype. qRT-PCR results confirmed that the wild genotype had higher expression levels (REL) in all 6 TF genes than the commercial genotype. Our transcriptomic analysis should help to unravel candidate genes that may be useful in the development of new drought-tolerant cultivars of this important tropical crop.

## Introduction

Drought stress is worldwide considered as the single most common limitation for agricultural production and represents a great threat that may lead to malnutrition and famine [1]. In plants, water deficit stress (WDS) results in a decreased leaf water potential, turgor loss, stomatal closure, cell death, and eventually plant death [2, 3]. Furthermore, drought stress induces changes on physiological and biochemical processes that affect plant growth, such as photosynthesis, respiration, translocation, ion uptake, carbohydrates and nutrient metabolism [2, 4]. Therefore, terrestrial plants have developed different adaptation strategies to deal with WDS [5, 6]. Those strategies include regulation of stomatal opening and optimization of the accumulation of protective proteins, sugars, metabolites such as proline, and antioxidants to maintain cell membrane stability, interrupting metabolic activity through dormancy during extended period of WDS, etc. [7]. Accordingly, under conditions of mild to moderate drought, drought-tolerant plant genotypes may have more flexible stomatal responses by maintaining longer periods of transpiration and CO_2_ assimilate more efficiently [7].

Papaya is a fruit of high worldwide consumption, thus, the global papaya production has grown significantly over the last few years due to its high nutritional value, excellent flavor, and its various uses in the food industry [8, 9]. Papaya is now ranked as the third most consumed tropical fruit [10]. Cultivated papaya has expanded to a greater extent in tropical and subtropical countries because it can be cultivated in highly diverse environments. However, this crop needs constant irrigation to achieve high productivity. Under water limiting conditions, commercial papaya genotypes may show 50% lower yields [11]. Populations of wild native *C*. *papaya* can be found in non-perturbed arid and semi-arid regions of the Yucatan Peninsula. These wild papayas have been naturally exposed to multiple environmental stresses such as drought and high temperatures in their natural environment. Wild papayas may have adapted to local stressful environmental conditions. However, the fruits from these wild papayas are small and of low quality so they are not marketable. On the contrary, commercial genotypes were developed to attain high fruit yields, but they are susceptible to abiotic stresses such as drought and heat.

In papaya, several traits characterize the differential response to drought stress; this response is controlled by a number of genes acting at molecular, cellular, and whole-plant level, to maintain water- and ion- homeostasis, and to protect plants from wilting, and thus ensuring survival under WDS conditions [12, 13]. In a recent study, [14] reported some biological processes regulated under moderate and severe drought stress in commercial papaya plants (Maradol Roja). However, these adaptive mechanisms derived from gene expression changes in signaling and transcription regulation, have not yet been documented in wild papaya plants under WDS. Therefore, in the present study we document a comparative transcriptomic analysis (RNA-seq) performed between a wild genotype with the commercial genotype (Maradol) under conditions of WDS. Understanding the molecular mechanisms that wild papaya genotypes may have to deal with abiotic stresses is important because they represent a genetic reservoir for abiotic stress tolerance genes, as they have evolved over centuries of continuous selection, developing different adaptation strategies to abiotic stresses, including drought. Thus, wild papayas are an excellent model system to study plant adaptation to drought stress as they hold a high potential, as genetic reservoirs, for the breeding of new stress-tolerant papayas.

In previous studies conducted by [15], transcription factors (TF) genes (MYB, bHLH, GRAS, NAC, and WRKY) were differentially expressed in two cultivars of *Camellia oleifera* exposed to different degree of drought stress. Other TF genes mitigating the effects of drought stress in plants [16] include DREB, MYB, WRKY, NAC and bZIP families [17–19]. The overexpression of some of those TF resulted in enhanced drought tolerance [20–22]. Our main interest is to understand the molecular responses to drought stress of wild papaya genotypes. In order to do so, we performed morphological and physiological analysis, and differential expression analysis of TF genes related to drought tolerance. The genetic pool found in papaya wild genotypes may assist breeding programs in the future to develop new cultivars of papaya with enhanced tolerance to drought stress.

Next-generation RNA-sequencing (RNA-seq) is a versatile platform with applications in many fields of plant biology [23, 24]. This method can help to identify expressed sequences in specific tissue at any specific time. It has been used to characterize differential expression or to study tissue-specific transcripts in responses to abiotic and biotic stresses [25–27]. In papaya, there are several transcriptomic studies in roots [28], flowers [29], fruit ripening [30, 31], and a transcriptome analysis of leaves, sap and roots of papaya plants under drought stress [14], however, as previously mentioned, up to date there are no reports on transcriptomic studies in response to WDS in wild papayas. Therefore, comparing transcriptomic changes occurring in both wild and commercial papaya genotypes in response to WDS is relevant, as it is necessary to understand the mechanisms involved in drought stress response of this tropical crop. Likewise, the identification of differentially expressed TF genes in papaya plants exposed to drought may provide further insight into the molecular mechanisms confering drought tolerance in this species.

Thus, the aim of the present study was to elucidate some of the physiological and molecular mechanisms that allow wild papayas to better tolerate WDS than their commercial counterparts. In order to do so, transcriptomic profiles (RNA-Seq) were constructed from leaves from two papaya genotypes of contrasting drought tolerance.

## Materials and methods

### Plant material and cultivation

Two *Carica papaya* genotypes: T (Wild tolerant genotype) (WA #30) and S (Commercial susceptible genotype) (cv. Maradol) were used. Fruits from these two genotypes were collected in Yucatan, Mexico (20°53’10” W 89°27’7” O and 20°53’19” W 89°21’13” O, respectively). Fruits were collected at their initial stage of physiological maturity (approximately 150 days after anthesis). 50 seeds extracted from fruits from each genotype were disinfected with 1% sodium hypochlorite for 5 min and rinsed thoroughly with distilled water. The seeds were given a pre-germinative treatment under greenhouse conditions, this process consisted in soaking the seeds in 200 mL of sterile distilled water for 24 h under constant stirring (for softening the testa). Subsequently, the seeds were placed on moistened flannels (previously sterilized) and placed in a germination chamber at 35°C. The resulting seedlings were placed in individual pots with substrate (peat moss and soil; 2:1 ratio) under greenhouse conditions (30 ± 2°C, relative humidity of 70% and maximum Photosynthetically Active Radiation (PAR) of 382 μmol photons m^-2^ s^-1^) where they were grown for 60 days until they were used for the experiment.

### Water deficit stress treatments

All plants from both genotypes were irrigated with 30 mL of distilled water every 2 days during the first 25 days, after that plants were watered with 50 mL every 2 days. A commercial foliar nutrient solution (Bayfolan® Forte Liquid, Bayer) (1mL L^-1^) was applied twice a week. Then, plants were randomly distributed into four different groups of 15 plants each, in a completely randomized block design, comprising 5 seedlings per each of 3 replicates (*n = 15*). The four treatments consisted in: 1) Seedlings from the susceptible genotype irrigated with 50 mL of water every two days during 14 days post-treatment (d.p.t.) (SW), 2) seedlings from the susceptible genotype subjected to WDS by withholding watering for 14 d.p.t (SD), 3) seedlings from the wild tolerant genotype irrigated with 50 mL of water every two days during 14 d.p.t (TW) and 4) seedlings from the wild tolerant genotype subjected to WDS by withholding water for 14 d.p.t. (TD).

### Physiological evaluation

To measure water potential (Ψ), a gardening puncher was used to get disks from fully expanded leaves (avoiding leaf midrib) from both genotypes when well-watered and during the WDS period. For each point,15 leaf disks (3 disks extracted from the same leaf per plant x 5 different plants) were used. Leaf disks (8-mm diameter) from all treatments were placed inside C-52 psychrometric chambers connected to a dew point hygrometer (Wescor HR-33T, Inc., Logan, Utah, USA). Samples were kept in the leaf chamber during 30 min before measuring the Ψ expressed in MPa.

Photosynthesis rates (Pn) were determined using a portable photosynthetic system (LI-COR LI-6400XT Inc., Lincoln, Nebraska, USA). Selected leaves were placed inside the leaf assimilation chamber (capacity of 6.25 cm^2^). The conditions within the chamber were set as follows: CO_2_ at a constant concentration of 400 μmol mol^-1^ (that was applied using compressed gas cylinders of CO_2_), air humidity in the chamber of 55%, a constant air flow of 200 μmol s^-1^, a flux density of photosynthetic photons of 382 μmol m^-2^ s^-1^ and a temperature inside the chamber of 23°C ±3°C.

To measure FV/Fm (efficiency of photosystem II; PSII), one leaf from each of the 5 replicates (the same leaf from which disks were later extracted for other measurements) were used. Leaves were enclosed in the leaf chamber of a fluorescence modulated systems analyzer (FMS2–Hansatech, Norfolk, UK). After a 20 min dark adaptation, the adaxial part of the leaf was exposed to a saturation pulse of 3000 μmol m^-2^ s^-1^ (100%) for 2 seconds. With these readings, the variable fluorescence quotient between variable fluorescence (Fv) over maximum fluorescence (Fm), (Fv/Fm), was determined, as reported by [32].

Electrolyte leakage (EL) expressed as percentage was calculated based on the protocols of [33–35]. Fifteen leaf disks (6 mm diameter) (3 disks from one leaf from each of the 5 replicate plants) were extracted and placed in 12 mL of distilled water. The disks were then incubated with constant shaking for 4 h on an orbital shaker. The initial conductivity (C1) was measured, then, disks were placed in an autoclave for 15 min, once the samples had cooled down (room temperature), the final conductivity (C2) was measured. EL was calculated according to the following equation: Electrolyte leakage = (C1 / C2) x 100%.

### Statistical analysis

To provide statistical support for physiological analyses, we use the Tukey multiple range test at *p<0.*05* for comparing treatments means with the Statgraphics Plus 5.1 Software (http://www.statgraphics.com). The graphs were performed using the Sigma Plot 11.0 software.

### Total RNA extraction and complementary DNA (cDNA) library preparation for sequencing

Seedlings of commercial-susceptible and wild-tolerant genotypes were subjected to water deficit stress (WDS) or well-watered treatment for 3, 7 and 14 days. Total RNA was isolated from 200 mg of fresh fully expanded leaves after treatment using a CTAB protocol as reported by [36]. The concentration and purity of RNA samples were determined by NanoDropTM 1000 Spectrophotometer (Thermo Scientific NanoDrop Technologies, LLC, Wilmington, DE, USA) and the quality was evaluated by agarose gel electrophoresis (1.5%) during 30 min at 80 V.

Three double stranded libraries for each one of the 4 treatments (SW, TW, SD and TD), that is a total of 12 libraries were built. However, in order to gain enough depth in the RNA-Seq analysis, we pooled together the 3 libraries made for each one of the four treatments, obtaining and sequencing only one sample per treatment. In order to obtain robust and accurate differential expression results we then employed the parametric bootstrap procedure proposed in [37]. The cDNA libraries were prepared using Illumina TruSeq RNA Sample Preparation Kit V2, according to the manufacturer´s manual (Illumina®, USA).

The resulting four cDNA libraries were subjected to purification using the AMPure XP beads (Beckman Coulter, Indianapolis, IN, United States) automated PCR purification system. The quality of cDNA libraries was tested using Agilent 2100 Bioanalyzer (Agilent, Santa Clara, CA, USA).

All the 4 cDNA libraries were sequenced using an *in-house* Illumina MiSeq instrument in pair-end read mode to generate reads of 300 base-paired long according to the manufacturer's instructions. Raw reads were checked for quality with FASTQC [38]. For each sequenced library, Poison random numbers were generated taking the observed raw expression as the Poisson parameter lambda, for each one of the genes at each library. The complete procedure was repeated 100 times, obtaining at each case the P values for each gene and contrast. The median of the P values in the 100 replicates was taken to represent P values for each gene. Median of the P values was transformed to Q values (False Discovery Rate; FDR). Fluorescent image processing, base-calling and quality value calculations for each of the three runs were performed using Illumina MiSeq^TM^ Control Software.

### *Carica papaya* transcriptome assembly, differential expression analysis & functional annotation

Raw sequence reads were checked for quality using FASTQC [38] and pre-processed to remove low quality reads (Q ≤ 30) (Table 1) using PRINSEQ software (v 0.20.4) [39]. Illumina adapters were identified and removed through a BLASTN alignment [40]. A *de novo* transcriptome assembly was performed using Trinity (v 20140717) software [41]. The sequences resulting from the assembly were filtered and selected based on length and GC content. The sequences with a length ≥ 300 nt and GC content ≥ 18 and ≤ 58 were selected for downstream analyses. These sequences were identified using BLAST [40] with an E-value cutoff of 1e^-03^ and High-scoring Segment Pair (HSP) coverage equal or greater than 20%. Two databases were used for identification of the transcripts: i) *Arabidopsis thaliana* proteome (TAIR v 10) [42] and ii) *C*. *papaya* proteome from NCBI (downloaded January 2016). Finally, a subset of assembled sequences was selected as *C*. *papaya* reference transcriptome based on length (≥ 300 nt), GC content (≥ 27%) and identification with a known protein product as described above. High-quality reads were aligned to *C*. *papaya* reference transcriptome using bowtie2 (v 2.2.6) [43]. Aligned reads were quantified using eXpress software (v 1.5.1) [44]. For each library, Poison pseudo-random numbers were generated taking the observed raw expression as the Poisson parameter lambda, for each one of the genes at each library. The complete procedure was repeated 100 times, obtaining in each case the P values for each gene and contrast. The median of the P values in the 100 replicates was taken to represent P values for each gene. Median of the P values was transformed to Q values (False Discovery Rate; FDR) [45].

**Table 1:** Libraries and sequences generated for the *Carica papaya* transcriptome.

ID	Treatment	Number of sequences	Number of filtered sequences (%)	Mean Length of sequences	Number of mapped reads to assembly (%)
**SW**	Maradol plants under optimal watering condition	11,526,981	5,684,671 (49.32%)	149.45	4,464,197 (78.53%)
**SD**	Maradol plants under WDS	10,477,772	6,252,509 (59.67%)	146.27	4,607,117 (73.68%)
**TW**	Wild plants under optimal watering condition	17,646,849	6,799,391 (38.53%)	131.88	5,443,440 (80.06%)
**TD**	Wild plants under WDS	13,115,633	7,841,307 (59.79%)	123.13	5,583,379 (71.21%)
	**Total**	**52,767,235**	**26,577,878**		**20,098,133**

SW: Susceptible genotype Watered; SD: Susceptible genotype Droughted; TW: Tolerant genotype Watered; TD: Tolerant genotype Droughted; WDS: Water Deficit Stress.

Differential expression analysis was performed when comparing commercial susceptible vs wild tolerant genotypes at 14 d.p.t. using edgeR from Bioconductor tools (v 2.10.9) [46, 47]. Differentially Expressed Genes (DEG) were identified with a fold change (FC) of 0.5 and a FDR corrected with p value <0.001. For up regulated genes a FC > 1.5 was defined, while for down regulated genes a FC ≤ 0.5 was defined, in both cases a FDR of p<0.001 was used. The DEG were functionally annotated using BLASTX [48] to the Plant Ref-Seq proteins from NCBI and with an E-value cut-off of ≤ 1e^−14^.

Blast2GO software was used to visualize the functional annotations [49] with the Gene Ontology (GO) and Enzyme Code (EC) terms. GO terms were assigned from the three main GO categories (molecular function, biological process and cellular component). The R statistical package (https://www.r-project.org) and R-Studio package (https://www.rstudio.com) were used to plot GO data into horizontal bar-plots and expression data into heatmaps using the default options of the package gplots. Sequencing data were deposited into SRA database under accession number (available on request).

### RT-qPCR analysis

Transcriptome sequencing data was validated by Real-Time quantitative PCR (qRT-PCR). Total RNA was extracted from a pool of three leaves from three biological replicates. Quantification of total RNA was done using Nano Drop^TM^ 1000 Spectrophotometer (Thermo Scientific NanoDrop Technologies, LLC, Wilmington, DE, USA). For first-strand cDNA synthesis, Superscript III reverse transcriptase was used, following the manufacturer’s protocol (Invitrogen/Life Technologies, CA, USA). The primer sequences were designed using Primer Express Software (Applied Biosystems, Foster City, CA, USA). In total, six selected candidate genes were evaluated by RT-qPCR in a thermocycler STEP ONE SYSTEM and StepOne Software ver. 2.3 (Applied Biosystems, Foster City, CA, USA). The primers pairs of the six candidate genes (CpHSF, CpMYB, CpNAC, CpNFY-A, CpERF, CpWRKY) and the reference gene (Elongation Factor 1-α; CpEF1α), are shown in S1 Table. The specificity of the reactions was confirmed by the standard melt curve method. CpEF1α gene was used as a reference gene to normalize all data. The 20 μL PCR reaction mixture was comprised of 2 μL of cDNA, 0.5 μL each of forward and reverse primers, 10 μL Syber-Green dye and 7 μL of ultrapure water, as indicated by [36]. Relative Expression Levels (REL) of target genes in different treatments was calculated using the 2^-ΔΔCT^ method. qRT-PCR conditions were as follows: 95°C for 10 min; 38 cycles of 95°C for 15 s, 60°C for 1 min. The melting curves were analyzed at 60^o^ to 95°C after 38 cycles. REL data were subjected to one-way analysis of variance (ANOVA) (*p*<0.05), compared using Tukey's test by Statgraphics Plus 5.1 Software (Statistical graphics Corp., USA).

## Results

### Morphological and physiological responses to WDS in both papaya genotypes

**Fig 1:**
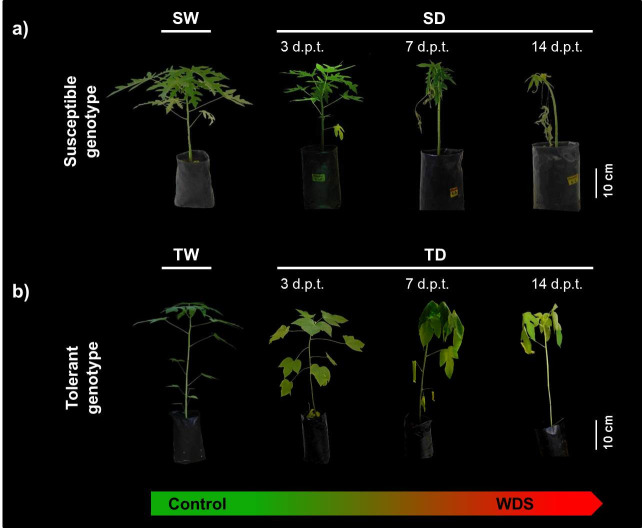
Morphological characterization of *C*. *papaya* plants under well-watered and WDS conditions. Representative photographs showing plants from the Susceptible (S) (a) and Tolerant (T) (b) genotypes, either well-watered (SW and TW), or during a 14 d.p.t. period of WDS (SD and TD). The time of stress exposure (from left to right; 3, 7 and 14 d.p.t. after water withholding) is indicated.

**Fig 2:**
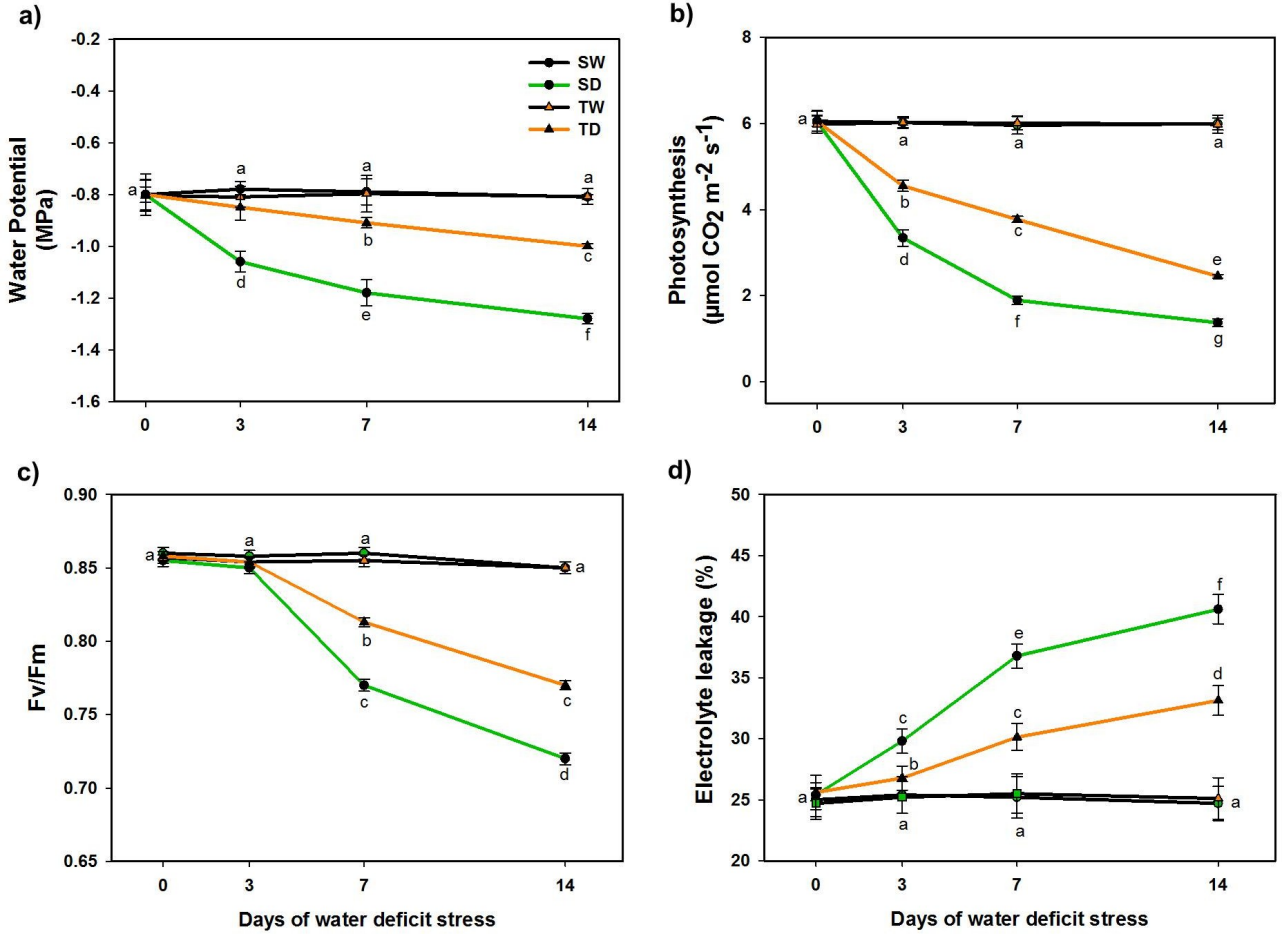
Measurement of physiological parameters in *C*. *papaya* plants under well-watered and WDS conditions. Water potential (MPa) (a), Photosynthesis (μmol CO_2_ m^-2^ s^-1^) (b), Fv/Fm (c) and Electrolyte leakage (%) (d) in plants irrigated daily (day 0, well-watered Control plants; SW and TW), and when plants were exposed to WDS for 3, 7 and 14 d.p.t. (SD and TD). Data are means ± SD of 3 replicates (n = 9). Different letters indicate significant differences (p<0.05).

A commercial susceptible (S) genotype and a wild tolerant (T) genotype were well-irrigated (SW and TW), or subjected to WDS (SD and TD) during 14 d.p.t. Under well-irrigated conditions, both genotypes (SW and TW) had a similar morphological development and comparable physiological performance. However, when exposed to WDS, the susceptible (SD) genotype showed greater leaf abscission, less turgid shoots and lower plant growth than the tolerant (TD) genotype (Fig 1). The susceptible (SD) genotype showed lower water potential (MPa), lower photosynthesis, lower Fv/Fm, as well as higher EL rates (Fig 2).

Under well-watered conditions, the susceptible and tolerant genotypes maintained Ψ values around -0.8 MPa. In contrast, when they were exposed to WDS for 14 d.p.t., the susceptible (SD) genotype reached Ψ values around -1.28 MPa, while the tolerant (TD) genotype reached Ψ values around -1.05 MPa (Fig 2A).

Under well-watered conditions both genotypes had similar photosynthetic rates (Pn) values around 6 μmol CO_2_ m^-2^ s^-1^. However, after 14 d.p.t. of water withholding, the susceptible (SD) genotype decreased Pn to values lower than 2 μmol CO_2_ m^-2^ s^-1^, while the tolerant (TD) genotype-maintained Pn values around 3 μmol CO_2_ m^-2^ s^-1^ (Fig 2B).

Under well-watered conditions, both genotypes maintained Fv/Fm values around 0.85. However, when exposed to WDS, the tolerant (T) genotype showed a 32% reduction of its Fv/Fm values (PSII efficiency), compared to the susceptible (S) genotype which showed higher reduction in their Fv/Fm values PSII (45%) (Fig 2C).

Regarding electrolyte leakage (EL; used as an estimation of membrane stability), under well-irrigated conditions both genotypes had an EL lower than 25%. Under WDS, however, the tolerant (TD) genotype maintained a lower EL (32%) than the susceptible (SD) genotype (that reached EL values higher than 40%), at the end of the 14 d.p.t. of WDS (Fig 2D).

### Sequencing, assembly and analysis of *C*. *papaya* reference transcriptome under WDS

Four cDNA libraries; for a tolerant and a susceptible genotype, either under optimal watering (SW and TW) and under 14 days of WDS (SD and TD) conditions, were constructed and sequenced utilizing Illumina technology to obtain a total of 52,767,235 reads (Table 1). Raw sequences were pre-processed to filter out, low quality reads and Illumina adapters, obtaining a total of 26,577,878 high quality reads (Table 1). A *de novo* transcriptome assembly was then performed to obtain a total of 135,459 contigs. The metrics of the assembly showed a N50 contig of 1,596 nt and an average contig size of 915 nt (Table 2). To remove redundancy from the assembly, the longest sequence (isoform) from each one of the “contigs” was selected resulting in 89,940 filtered contigs or singletons.

**Table 2:** Metrics of the *de novo* transcriptome assembly of *Carica papaya*.

Metric	Raw Contigs	Filtered Contigs
N50 contig (bp)	1,596	1,785
Average size of contigs (bp)	915	1,340
Minimum length (bp)	201	300
Maximum length (bp)	24,234	24,234
GC content (%)	≥ 18%	≥ 27%
Total transcript number	**135,459**	**29,070**

These singletons sequences were annotated by function using BLAST [39], using the *A*. *thaliana* proteome [42] and the *C*. *papaya* proteome from NCBI as references databases. Finally, 29,070 transcripts that were identified by BLAST (E-value ≤ 1e^-03^; HSP ≥ 20%) with a length ≥ 300 nt (N50 contig of 1,785 nt; average contig size of 1,340 nt) and GC content ≥ 27% were selected for downstream analysis (Table 2). Hereafter, the 29,070 assembled and fully identified transcripts will be referred to as the “Reference Papaya Transcriptome” (RPT).

**Fig 3:**
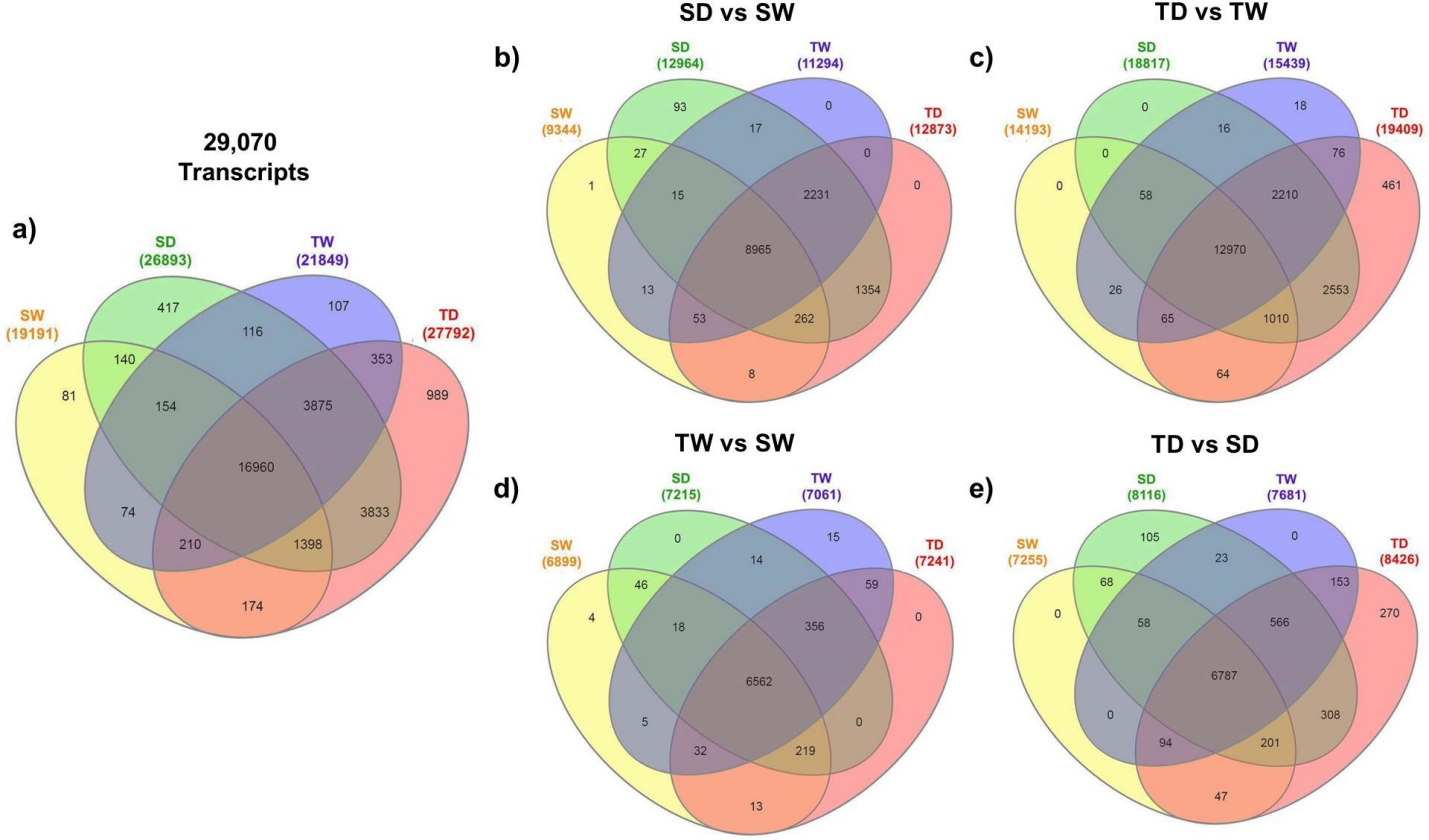
Venn diagram for differentially expressed genes in both papaya genotypes under well-watered or WDS conditions. Genes of reference transcriptome expressed in both genotypes under well-watered (SW and TW) or after they were subjected to WDS for 14 d.p.t. (SD and TD) **(a).** DEGs obtained for: SD vs SW **(b)**, TD vs TW **(c)**, TW vs SW **(d)** and TD vs SD **(e)**. Numbers in each intersection represent the number of detected genes with at least one read (gene tag) in these intersections.

A transcriptome expression analysis for all the four treatments (SW, SD, TW and TD) showed that 16,960 genes (58.34%) were expressed. Interestingly, the analysis also revealed that approximately 989 genes (3.4%) were expressed only in the tolerant (TD) wild genotype. In contrast, only 417 genes (1.43%) were expressed only in the susceptible (SD) genotype (Fig 3A). To identify differentially expressed genes (DEG), filtered reads derived from susceptible and tolerant genotypes under optimal (SW, TW) and under WDS (SD, TD) conditions were aligned to RPT and compared between genotypes (Table 1). Statistical analysis for mapped reads was performed to compare treatments: i) SD vs. SW; ii) TD vs. TW; iii) TW vs. SW, iv) TD vs. SD. Results showed that when comparing SD vs. SW, 13,040 genes (44.8%) were identified as differentially expressed (DE) (Fig 3B); 19,528 genes (67.1%) when comparing TD vs. TW, (Fig 3C); 7,344 genes (25.2%) when comparing TW vs. SW (Fig 3D) and 8,681 genes (29.8%) when comparing TD vs. SD (Fig 3E).

Interestingly, when comparing the susceptible genotypes under WDS against optimal watering treatments (SD vs. SW), a total of 89 genes were differentially expressed only in the susceptible (SD) genotype (Fig 3B). On the contrary, comparing the tolerant genotype exposed to WDS against optimal watering (TD vs. TW), 461 genes were differentially expressed in the tolerant (TD) genotype exposed to WDS (Fig 3C). When both genotypes were compared when they were under optimal watering conditions (TW vs. SW), 4 genes were expressed only in the susceptible genotype (SW) while 15 genes were expressed only in the tolerant one (TW) (Fig 3D).

Finally, when both genotypes were compared under WDS (TD vs. SD), just 105 DEGs were expressed only in the susceptible genotype (SD), but 270 DEGs were expressed only in the tolerant genotype (TD) (Fig 3E).

**Fig 4:**
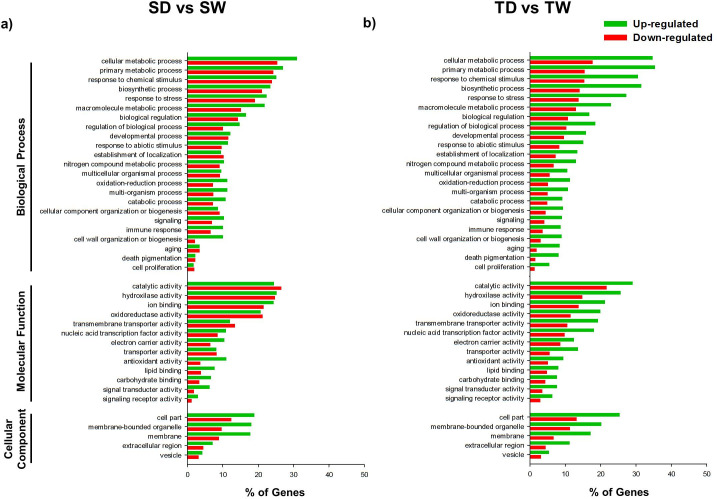
Gene Ontology functional annotation of DEGs in SD vs SW and TD vs TW treatments. GO Slim terms for Biological Process, Molecular Function, and Cellular Component assigned to up-regulated and down-regulated genes (%) found in (a) the susceptible *C*. *papaya* genotype under WDS for 14 d.p.t. compared to well-watered (SD vs SW). and (b) the wild tolerant *C*. *papaya* genotype under WDS for 14 d.p.t. compared to well-watered treatment (TD vs TW). GO associations were assigned by a BLASTX search against *A*. *thaliana* proteome.

**Fig 5:**
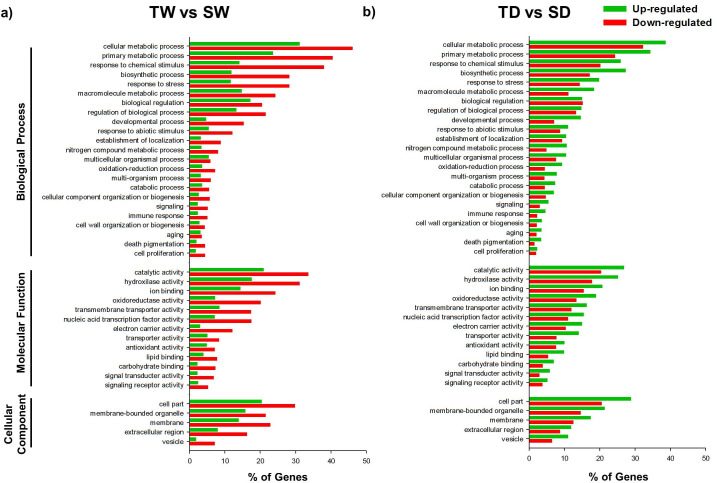
Gene Ontology Functional annotation of DEGs in TW vs SW and TD vs SD treatments. GO Slim terms for Biological Process, Molecular Function, and Cellular Component assigned to up-regulated and down-regulated genes (%) from both *C*. *papaya* genotypes, when (**a**) well-watered (TW vs SW); or (**b**) when both genotypes were subjected to WDS for 14 d.p.t. (TD vs SD). GO associations were assigned by a BLASTX search against *A*. *thaliana* proteome.

The analysis of the three main gene ontology (GO) categories (Biological process, Molecular function and Cellular component) revealed a total of 41 subcategories for up- and down-regulated genes (Figs 4 and 5). Comparing the S genotype when exposed to WDS vs. when it was maintained under well-watering conditions (SD vs. SW), a greater number of up-regulated genes was observed under WDS in some categories. However, this was not the case for various subcategories (13%) of the Biological process category (establishment of localization, cellular component organization or biogenesis, cell proliferation subcategories), as well as for various subcategories (23%) of the Molecular function category (i.e catalytic activity, oxidoreductase activity, transmembrane transporter activity) (Fig 4A).

On the contrary, the wild tolerant genotype (T) showed a greater and more notorious number of up-regulated genes for all the GO category (Biological process, Molecular function and Cellular component), when exposed to WDS than when grown under optimal watering conditions (TD vs.TW) (Fig 4B).

When both genotypes were compared both under optimal conditions (TW vs SW), the tolerant genotype showed a higher number of down-regulated transcripts for all GO categories es than those shown by the susceptible genotype (Fig 5A).

However, when comparing both genotypes both under WDS conditions (TD vs. SD), the wild tolerant genotype showed a greater number of up-regulated genes in all 41 GO categories (Biological process, Molecular function and Cellular component) and their subcategories (Fig 5B).

### Identification of differentially expressed transcription factors related to WDS

**Fig 6:**
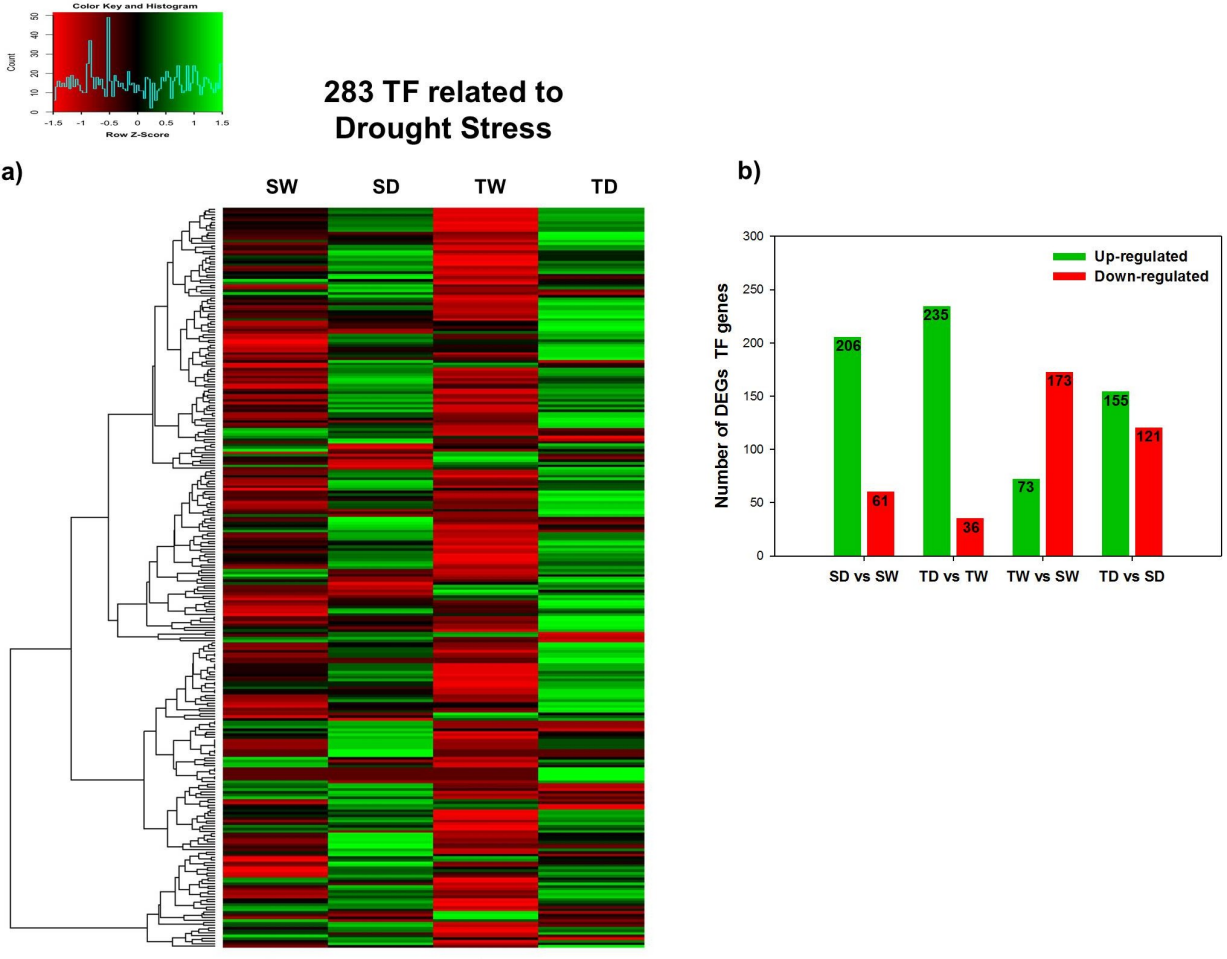
DEGs analysis in both *C*. *papaya* genotypes under well-watered or WDS conditions. Hierarchical clustering of 283 transcripts of TF genes identified in the papaya transcriptome of both genotypes when well-watered (SW and TW) or when exposed to WDS for 14 d.p.t. (SD and TD). Red indicates down-regulated, green indicates up-regulated and black unchanged values, as shown on the color scale at the side of the figure **a**). Comparison of the DEGs number of 283 TF genes evaluated by up- and down-regulated in the papaya transcriptome of both genotypes, when well-watered (SW and TW) or when they were exposed to WDS for 14 d.p.t. (SD and TD) **b)**.

A data-mining analysis of the DEGs revealed 283 TF related to drought stress (Fig 6; S2 Table). These TF are classified into 13 major families: 1) DREB (24 genes); 2) bZIP (15 genes); 3) HSF (12 genes); 4) MYC (4 genes); 5) MYB (43 genes); 6) NAC (28 genes); 7) AP2 (10 genes); 8) NFY (15 genes); 9) ERF (16 genes); 10) WRKY (29 genes); 11) C_2_H_2_ (30 genes); 12) bHLH (47 genes); 13) ERD (10 genes) (S2 Table). A heatmap plot of these 283 TF revealed different expression pattern among both genotypes. Under well-watered control plants (SW and TW) both genotypes showed similar expression profiles, both genotypes had a large number of down-regulated TF genes. In contrast, when exposed to WDS, plants (SD and TD) the T genotype showed in general a higher number of up-regulated TF genes than S genotype (Fig 6A).

DEGs analysis revealed that from the 283 TF transcripts found, 206 were up-regulated while 61 were down-regulated in the susceptible genotype under WDS versus optimal watering conditions (SD vs SW). Likewise, 235 TF transcripts were up-regulated while 36 were down-regulated for the tolerant genotype under WDS versus optimal watering conditions (TD vs TW). When well-watered control plants (SW and TW) were compared, the genotype tolerant showed a larger number of down-regulated TF genes (173) and few up-regulated TF genes (73). However, when comparing genotypes under WDS conditions (TD vs. SD), the tolerant genotype showed 155 up-regulated TF genes and 121 down-regulated TF genes (Fig 6B; S2 Table). Moreover, 19 TF genes were identified as being up-regulated in the tolerant (TD) genotype (TD vs TW), but in the susceptible (SD) genotype (SD vs SW), these genes were down-regulated (Table 3).

**Table 3:** TF genes associated to drought stress, that showed up-regulated differential expression in plants of the wild tolerant genotype exposed to 14 d.p.t. of WDS vs well-watered plants (TD vs TW), but down-regulated differential expression in the commercial susceptible genotype plants exposed to 14 d.p.t. of WDS vs well-watered plants (SD vs SW).

No.	Family	Seq_name	Short_description	FC (SD vs SW)	FC (TD vs TW)
**1**	MYC	c18383_g1_i1	ByTAIR: N-MYC downregulated-like 2	0	3
**2**	MYB	c24860_g1_i1	ByTAIR: myb domain protein 14	0	2
**3**	MYB	c18414_g1_i2	ByTAIR: myb domain protein 62	0	17
**4**	MYB	c22708_g1_i1	ByTAIR: myb domain protein 7	1	3
**5**	NAC	c21435_g1_i2	ByTAIR: NAC domain containing protein	0	2
**6**	NAC	c57862_g1_i1	ByTAIR: NAC domain containing protein 83	0	2
**7**	AP2	c26623_g1_i1	ByTAIR: related to AP2.7 transcriptional factor	1	3
**8**	WRKY	c1593_g1_i1	ByTAIR: WRKY DNA-binding protein 27	0	2
**9**	WRKY	c20181_g1_i1	ByTAIR: WRKY DNA-binding protein 49	0	6
**10**	WRKY	c16347_g1_i2	ByTAIR: WRKY DNA-binding protein 51	0	21
**11**	WRKY	c82476_g1_i1	ByTAIR: WRKY DNA-binding protein 55	0	2
**12**	C2H2	c69592_g1_i1	ByTAIR: C2H2 and C2HC zinc fingers protein	0	5
**13**	C2H2	c31341_g1_i1	ByTAIR: C2H2-type zinc finger family protein	1	2
**14**	C2H2	c32531_g1_i1	ByTAIR: C2H2-type zinc finger family protein	0	3
**15**	bHLH	c18515_g1_i1	ByTAIR: basic helix-loop-helix (bHLH)	0	2
**16**	bHLH	c29432_g1_i3	ByTAIR: basic helix-loop-helix (bHLH)	1	2
**17**	bHLH	c9326_g1_i1	ByTAIR: basic helix-loop-helix (bHLH)	0	3
**18**	ERD	c25503_g1_i3	ByTAIR: ERD (early response to dehydration)	0	5
**19**	ERD	c86565_g1_i1	ByTAIR: ERD (early-responsive to dehydration)	0	3

**Fig 7:**
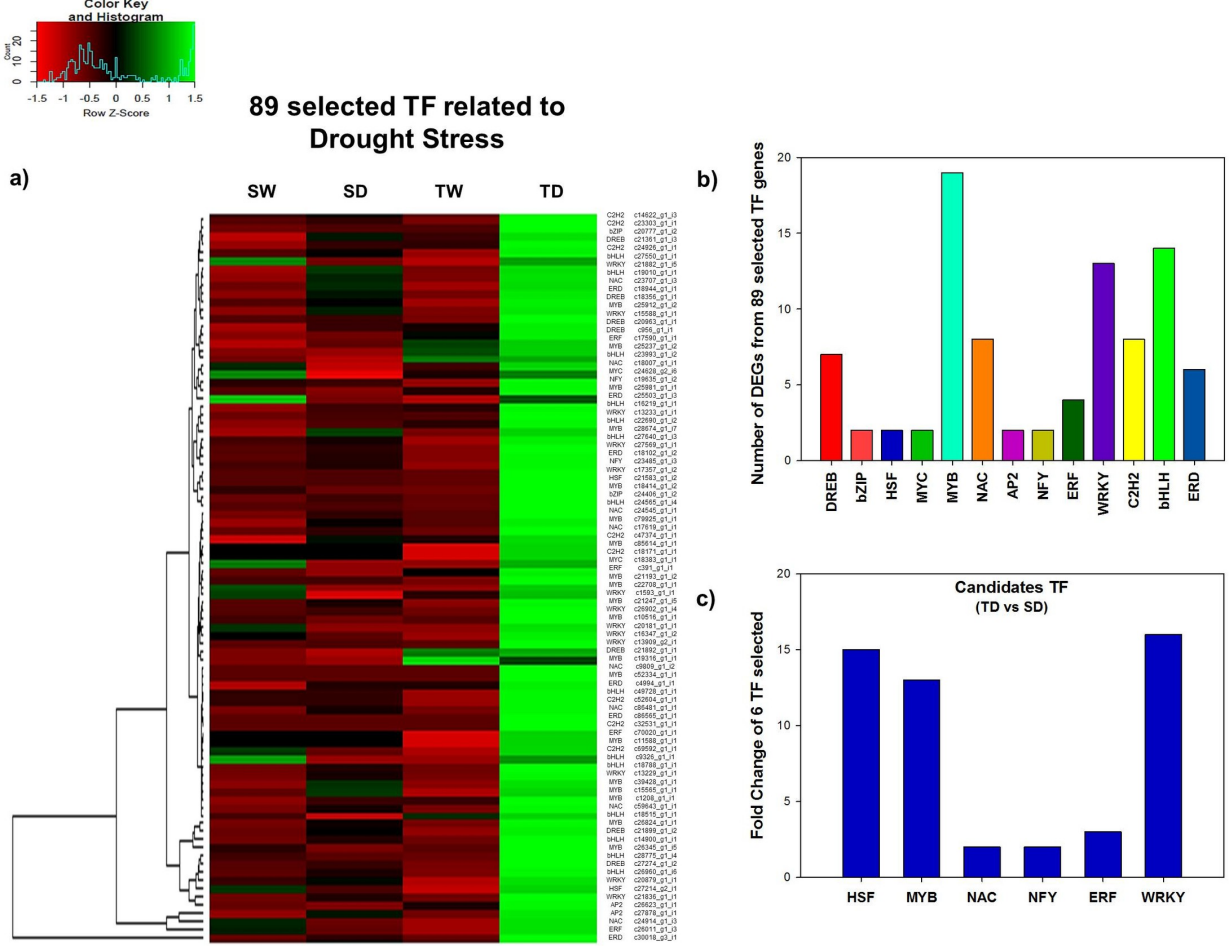
DEGs analysis of 89 TF genes related to drought stress, selected according to their high number of transcripts per million (TPM) and high fold change rate. Hierarchical clustering of 89 TF genes identified in the papaya transcriptome, based on the ratio between SD/TD, with fold change rates greater than 1.5 (*p < 0*.*001*). Red indicates down-regulated, green indicates up-regulated and black unchanged values, as shown on the color scale at the side of the figure **a**). Distribution by TF family of the number of DEGs from the 89 selected TF genes **b)**. Fold Change of TF candidate genes selected, according to the ratio between SD/TD, to be validated by RT-qPCR **c)**.

Additionally, the top 89 highly expressed TF genes were identified and selected, based on a fold change greater than 1.5, between the treatment TD vs SD (S3 Table). The expression analysis for these 89 TF in all four conditions displayed strong evidence that under WDS, most of these TF are highly expressed in the wild tolerant genotype (TD) but less expressed in the susceptible genotype (SD) (Fig 7A). The 89 selected TF genes were classified into 13 gene families, the number of differentially expressed genes (DEGs) found in each family is shown in parenthesis: MYB (19 genes), bHLH (14 genes), WRKY (13 genes), C2H2 (8 genes), NAC (8 genes), DREB (7 genes), ERD (6 genes), ERF (4 genes), bZIP(2 genes), HSF (2 genes), MYC (2 genes), AP2 (2 genes) and NFY (2 genes) (Fig 7B; S3 Table).

### RT-qPCR analysis of 6 selected TF associated to drought responses

Based on their high fold change, 6 TF were selected to undertake RT-qPCR analysis: c21583_g1_i2 contig (CpHSF family with FC of 15 in TD/SD), c18414_g1_i2 contig (CpMYB family with FC of 13 in TD/SD), c24914_g1_i3 contig (CpNAC family with FC of 2 in TD/SD), c23485_g1_i3 contig (CpNFY-A family with FC of 2 in TD/SD), c26011_g1_i3 contig (CpERF family with FC of 3 in TD/SD) and c17357_g1_i2 contig (CpWRKY family with FC of 16 in TD/SD) (Fig 7C).

**Fig 8:**
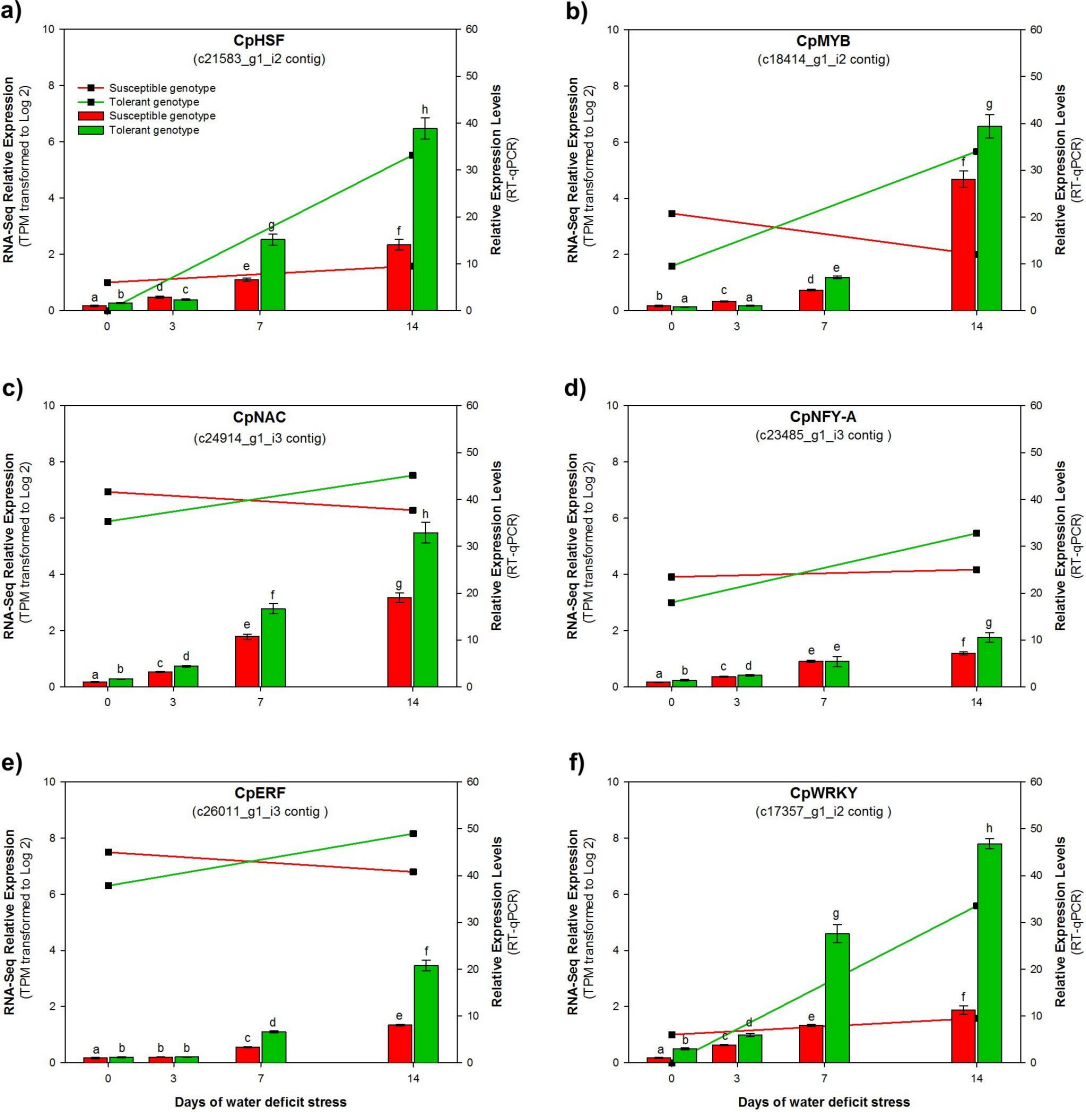
Expression levels of six selected TF candidate genes from leaves of the two *C*. *papaya* genotypes when well-irrigated (day 0), or when exposed to 14 d.p.t. of WDS. Red (S) and green (T) lines represent the expression level of transcripts per million (TPM) of the following 6 TF genes: CpHSF **a**), CpMYB **b**), CpNAC **c**), CpNFY-A **d**), CpERF **e**) and CpWRKY **f**). In the case of TPM data were transformed to Log 2 values. The red (S) and green (T) bars represent the Relative Expression Levels (REL) of the same genes determined by RT-qPCR, after 3, 7 and 14 of WDS. REL data are the means ± SD of three biological replicates. Different letters indicate significant differences.

These six candidate genes were validated through RT-qPCR analysis. The results confirmed that under optimal watering conditions, those TF had a low Relative Expression Levels (REL) in both genotypes. However, the exposure to WDS was able to induce the expression of those TF after just 3 days of exposure. As WDS developed, the expression levels of those TF increased, particularly at the tolerant (TD) genotype. At day 14 of WDS, REL values for all CpHSF, CpMYB, CpNAC, CpNFY-A, CpERF and CpWRKY genes showed significant differences between genotypes, in all cases, the tolerant (TD) genotype showed significantly higher REL values than the susceptible (SD) genotype (Fig 8). For CpHSF, T genotype reached significantly higher REL values than S genotype (39 vs. 14) (Fig 8A). For CpMYB, T genotype reached significantly higher REL values than S genotype (39 vs. 28) (Fig 8B). In the case of CpNAC, T genotype reached significantly higher REL values than the S genotype (33 vs. 19) (Fig 8C). In the case of CpNFY-A, T genotype reached significantly higher REL values than S genotype (11 vs. 7) (Fig 8D). For CpERF, T genotype showed higher REL values tan S genotype (21 vs.8) (Fig 8E). Regarding CpWRKY, T genotype showed significantly higher REL values than S genotype (47 vs. 11) (Fig 8F).

In general, the measured patterns of expression by RT-qPCR for these selected TF for both genotypes when well-watered and after 14 days of WDS, followed closely those expression levels (transcripts per million; TPM) predicted by the transcriptome analysis for those TF genes (shown as log 2 of TPM; plotted as lines in Fig 8).

## Discussion

Commercial papaya is considered drought susceptible as, during dry periods, irrigation is necessary to increase their growth and fruit production [50]. In our study, WDS lead to decrease in various physiological parameters in the commercial genotype, as previously reported [50, 51]. When the susceptible and tolerant genotypes were well-watered, both genotypes maintained Ψ values around -0.8 MPa, similar to those observed by [52] in well-irrigated papaya cv. Baixinho. After 14 days of WDS, the susceptible genotype reached values as low as -1.28 MPa, while the wild genotype reached -1.05 MPa during the same stress period. Similar water potential values have been reported from papaya plants exposed to long periods of WDS [52]. The photosynthetic rates found in this study are similar to Pn values in other studies [53], we report photosynthetic rates between 8–10 μmol CO_2_ m^-2^ s^-1^ for *C*. *papaya* plants under irrigation for both genotypes, but again under WDS, the wild tolerant genotype was able to maintain higher Pn values than the commercial one at the end of the 14 days of WDS. The analysis of chlorophyll fluorescence emission from photosystem II (PS II) is useful to characterize the effects of different types of environmental stress. Several authors agree that Fv/Fm is a very sensitive physiological parameter that can be used as an effective quantitative measurement to characterize the magnitude of stress to which a plant is exposed [54]. Similar Fv/Fm values to those measured in our experiment were reported earlier in other genotypes exposed to WDS [55] and again the wild tolerant genotype was able to maintain higher efficiency of PSII than the commercial one, after the same WDS period. On the other hand, it is well known that an increase in Electrolyte Leakage (EL) normally indicates the degree of damage to the membrane by heat, salinity or WDS [33]. Likewise, it has been proposed that a drought tolerant plant may maintain its membrane integrity (showing reduced EL) even when subjected to stress condition [56]. In our experiment, the results suggest that under WDS conditions, the wild tolerant (TD) genotype has a more effective mechanism to avoid membrane damage, than the susceptible (SD) genotype. The results of the physiological measurements confirmed the visual morphological assessment, indicating that the wild tolerant (TD) genotype was effectively more tolerant to WDS than the susceptible (SD) genotype.

Comparison of transcriptome profiling in wild- and commercial-papaya genotypes allowed a deeper insight into the complexity of plant response to drought stress at the molecular level. Drought stress mediated by gene expression has been sparsely studied in wild species. The transcriptome sequences data obtained in our study can be a valuable genetic resource to understand the molecular responses to drought in wild papaya. It is important to note that this is the first transcriptomic study including wild-papayas genotypes. A comparative analysis of DEGs revealed that under WDS wild papaya plants have a larger number of DEGs than under optimal watering conditions. These results correlate with the greater number of DEGs (19,528) observed in the tolerant genotype, and strongly suggest that wild papayas are an important reservoir for stress tolerance genes. Similarly, transcriptomic profiles from *Cucumis sativus* L. plants exposed to WDS indicated a significant increase of genes (especially in the metabolic process, membrane, and catalytic activity) when different *Cucumis sativus* L. cultivars were exposed to drought during 4 d.p.t. [57]. Thus, our results suggest that during WDS, a large number of genes related to Biological process, Molecular function and Cellular component increase in function of drought stress for wild papaya, in this way, this tolerant genotype can better adapt to WDS. Similarly, in other plant species a high enrichment of DEG in plants exposed to WDS was observed [58, 59]. Interestingly, we detected the expression of nucleic acid transcription factor activity genes, another interesting group of genes involved in drought stress.

TF are proteins that can activate or suppress the transcription of downstream target genes by binding directly to promoters of target genes in a sequence-specific mode [60]. Thus, TF are considered candidate genes capable of regulating gene expression in response to environmental and physiological signals [61, 62]. Therefore, identification and evaluation of TF genes related to stress tolerance are essential for molecular enhancement in papaya breeding programs [63]. For instance, bHLH played important roles in ABA-signaling and abiotic stress response in *Camellia sinensis* [64], NAC and C2H2 zinc finger, have been reported in responses to various abiotic stresses in *Z*. *mays* [65], WRKY have shown to be up regulated in response to drought stress in *Z*. *mays* [66], and MYB binding site (CAACTG) is involved in drought-stress regulation [67]. Similarly, it that AP2/ERF (Apetala 2/Ethylene Response Factor) and ERD (Early Response to Dehydration) have responded, because DREB (Dehydration Responsive Element Binding proteins) and ERF (Ethylene Response Factor) are two subgroups of the AP2/ERF that are involved in regulating stress-related genes in an ABA-independent manner, by interacting with DRE sequences (Table 3) [68]. Our results are in line with similar studies, in bitter apple (*Citrullus colocynthis* L.), where transcriptome analysis showed that leaves gene expression was significantly altered when the plants were exposed to drought stress [69]. In rice (*Oryza sativa* L.) transcriptome, a species of high food importance, a total of 36 differentially expressed TF genes were found between a tolerant and a sensitive line, under drought stress. Among them, AP2/EREBP, MYB, bHLH and NAC genes were significantly up-regulated in the tolerant line (H471) under drought [70]. [13] reported 18,369 expressed genes in lentil (*Lens ulinaris* Medikus) in response to drought stress using next generation sequencing; among the identified DEGs, they found R2R3 family MYB, AP2/ERF like family, WRKY group II and III families and bZIP. Recently in *Zea mays*, [27] performed a RNA-seq analysis finding a total of 251,145 transcripts where they found 532 up-regulated genes and 83 down-regulated genes in response to drought. Likewise, a transcriptome study performed in two barley genotypes with different level of drought tolerance [71] revealed 1802 DEGs identified in leaves and roots. In our study with papaya, similar families of TF were found when plants were exposed to WDS. Interestingly, [14] analyzed a transcriptomic profile from commercial C. papaya plants exposed to mild and severe drought. Thus, through co-expression network analysis, reported 17 stress-related TFs in leaves and roots. Through this analysis (co-expression network), it was reported that TF genes such as WRKY70, MYB94, RAP2.11, bHLH, HSFB-2A and AP2/ERF showed the highest degree of distribution in leaf and root. Also, TF genes such as RAP2.6, bHLH, MYB48, MYB94, bZIP1 and WRKY75 were shared between the leaf and root by co-expression network analysis. In our study, we also found TF genes (MYB, AP2.7, WRKY, bHLH; Table 3) that showed up-regulated differential expression in our tolerant genotype (wild). Likewise, TF genes as HSP, MYB, NAC, NFY-A, ERF and WRKY (Fig 8) were selected in our study based on high fold change and expression. However, these 6 TF genes selected by us, are not the same genes that [14] reported, although some of them are for the same TF genes families (WRKY, MYB, bHLH, AP2/ERF) that regulates abiotic stress responses in plants. Our present results confirm that those TF are important in response to WDS, but add the fact that wild *C papaya* plants collected from their center of origin are more responsive to WDS than the commercial papaya genotype.

Comparing the estimated expression levels by RNA-Seq and RT-qPCR, our results indicate that tolerant genotype have higher REL than the susceptible genotype for CpHSF, CpMYB, CpNAC, CpNFY-A, CpERF and CpWRKY genes after 14 d.p.t. of WDS (Fig 8A–8F). [72] reported that DREB, ERF, MYB, NAC and WRKY TF genes play an important role in engineering drought tolerance in transgenic plants. According to the study, in *Glycine max* L. Merr, 8 NAC genes (GmNAC004, GmNAC021, GmNAC065, GmNAC066, GmNAC073, GmNAC082, GmNAC083 and GmNAC087) related to drought stress, showed greater transcript levels in the drought-tolerant soybean varieties than in the drought-sensitive varieties [73]. In this context, our results in papaya indicate that CpNAC gene also showed greater expression levels in tolerant (TD) genotype than the susceptible (SD) genotype, when they were exposed to WDS. Regarding the WRKY gene, the high expression levels of CpWRKY gene validated by RNA-Seq and RT-qPCR, confirms that this TF could play an important role in the response of papaya to drought stress. Such homologous of genes (OsWRKY13) regulates the expression of more than 500 stress-responsive genes in rice [74].

Our quantitative results using RT-qPCR are consistent with those predicted from the RNA-Seq analysis. Thus, we confirmed that when plants from both papaya genotypes were subjected to WDS, the expression of important TF genes associated to drought-response was induced, but they were most abundantly expressed in the wild tolerant genotype.

We are now investigating if important drought-tolerance genes might have been lost during the papaya domestication process. The knowledge gained from this study, should pave the way to an improved understanding of the molecular processes involved in the papaya response to water deficit stress, and they might set the basis for papaya wild-relative-based plant breeding programs, aiming to develop new papaya cultivars with enhanced drought tolerance.

## Conclusions

The present study confirmed that the papaya wild genotype is physiologically more drought-tolerant than the commercial papaya genotype. The superior drought tolerance of the wild genotype may have resulted from its ability to maintain lower EL (that might reflect its ability to maintain membrane stability), higher Fv/FM values (that may reflect its higher efficiency of PSII) and higher stomatal conductance (that might reflect that their stomata remained open longer) than its commercial counterpart when subjected to WDS.

Furthermore, our detailed transcriptome analysis indicated that the wild genotype has a higher number of up-regulated DEGs than the commercial genotype when exposed to 14 d.p.t. of WDS. Likewise, wild genotype consistently showed higher expression levels of TF genes, and higher expression levels of drought-associated TF, than the commercial genotype. Therefore, that improved performance of the wild genotype under WDS, may be related to its ability to maintain high expression levels of key TF genes that may in turn, regulate other downstream genes involved in triggering protective mechanisms to reduce WDS-related damage and they should be part of the complex molecular mechanism for drought tolerance in this tropical species. Although more research is needed, the high expression levels showed by these 4 TF genes in response to WDS, observed in the transcriptome analysis and confirmed with the qRT-PCR data, suggest that these 4 TF (CpWRKY, CpHSF, CpMYB and CpNAC) might be important candidate TF genes involved in drought tolerance in this species, that might be useful in breeding programs aiming to generate cultivars with enhanced drought tolerance.

## Supporting information

S1 Table Excel file containing primers pairs used for RT-qPCR analysis. (XLSX)

S2 Table Excel file containing 283 DEGs of TF type. (XLSX)

S3 Table Excel file containing 89 selected TF type based on their TPM content and fold change >1.5. (XLSX)
